# Deubiquitinating Enzyme-Mediated Signaling Networks in Cancer Stem Cells

**DOI:** 10.3390/cancers12113253

**Published:** 2020-11-04

**Authors:** Kamini Kaushal, Suresh Ramakrishna

**Affiliations:** 1 Graduate School of Biomedical Science and Engineering, Hanyang University, Seoul 04763, Korea; kamini27@hmail.hanyang.ac.kr; 2 College of Medicine, Hanyang University, Seoul 04763, Korea

**Keywords:** cancer stem cells, deubiquitinating enzymes, DUB inhibitors, signaling, stemness

## Abstract

**Simple Summary:**

Cancer stem cells (CSCs) have both the capacity for self-renewal and the potential to differentiate and contribute to multiple tumor properties. The function of CSCs can be regulated by well-balanced process of ubiquitination and deubiquitination of proteins related to the specific stemness of the cells executing various stem cell fate choices. Growing evidence suggests that the involvement of deubiquitinating enzymes (DUBs) in altering several signaling pathways leading to survival of CSCs. In this review, we have compiled all the evidences of DUBs and summarized its role in regulating several signaling network in cancer stem cells.

**Abstract:**

Cancer stem cells (CSCs) have both the capacity for self-renewal and the potential to differentiate and contribute to multiple tumor properties, such as recurrence, metastasis, heterogeneity, multidrug resistance, and radiation resistance. Thus, CSCs are considered to be promising therapeutic targets for cancer therapy. The function of CSCs can be regulated by ubiquitination and deubiquitination of proteins related to the specific stemness of the cells executing various stem cell fate choices. To regulate the balance between ubiquitination and deubiquitination processes, the disassembly of ubiquitin chains from specific substrates by deubiquitinating enzymes (DUBs) is crucial. Several key developmental and signaling pathways have been shown to play essential roles in this regulation. Growing evidence suggests that overactive or abnormal signaling within and among these pathways may contribute to the survival of CSCs. These signaling pathways have been experimentally shown to mediate various stem cell properties, such as self-renewal, cell fate decisions, survival, proliferation, and differentiation. In this review, we focus on the DUBs involved in CSCs signaling pathways, which are vital in regulating their stem-cell fate determination.

## 1. Introduction

Signal transduction is the cellular response to a physical or chemical signal and is mediated by a cascade of molecular events initiated when a particular ligand binds to its corresponding receptor, known as activation of a signaling pathway. Various cellular signaling pathways interact with one another and form networks that coordinate cellular responses through combinatorial signaling events. These responses include variations in transcriptional, translational, and post-translational activities at the molecular level, which can either change the conformation of proteins or their subcellular locations and ultimately influence cellular mechanisms.

Stem cells in multicellular organisms are defined as cells having the ability to self-renew or differentiate into various types of cells and proliferate to generate more of the same stem cells [1]. Stem cells are categorized into three types: embryonic stem cells (ESCs), adult stem cells (ASCs), and induced pluripotent stem cells (iPSCs). ESCs are derived from the inner cell mass of the blastocyst and can proliferate and differentiate without limits. ASCs also have the capacity for self-renewal, but they occur in an already differentiated tissue and can only differentiate into types of the source tissue [2]. iPSCs are generated from adult somatic cells by reprogramming them with defined transcriptional factors [3].

Mostly, stem cells use their capacity for unlimited proliferation to produce differentiated cells. However, recent evidence indicates that differentiated cells can de-differentiate into stem cells with phenotypic plasticity [4]. Generally, stem cell de-differentiation occurs during tissue regeneration. However, that process can be blocked by negative feedback from differentiated cells to maintain cell homeostasis [4].

Tumors can originate from normal stem cells and acquire the hierarchical characteristic of normal tissue. Therefore, tumors are assumed to be maintained by so-called cancer stem cells (CSCs), which are similar to ASCs in their ability to self-renew and generate tumor cells indefinitely. CSCs can self-renew through the asymmetric division of cells to differentiate into a non-tumorigenic bulk tumor mass [5]. In other words, CSCs divide to form either two CSCs or one CSC and one daughter cell. When CSCs isolated from an original tissue were transplanted into mice with severe combined immunodeficiency disease (SCID), a new tumor was formed [6]. Recent evidence indicates that even tumor cell populations undergo de-differentiation, which increases cell proliferation, tumor progression, and treatment resistance by increasing the growth rate of the tumor stem-cell population [7]. Oncogenic mutations in ASCs could explain the initiation and formation of cancers in the intestines, skin, and other specialized systems [8,9,10]. Mutations in highly differentiated cells also can give rise to CSCs that can self-renew indefinitely [11]. 

Signaling pathways, such as Notch, Wnt, and Sonic hedgehog (Shh), are associated with normal stem cell development and regulation of CSCs, and their dysregulation can cause cancer. The growth of CSCs can be controlled by the phosphatase and tensin homolog (PTEN) and signaling molecules from the polycomb family. The precise regulation of those pathways through ubiquitination/deubiquitination activities of regulatory proteins is crucial for the proper execution of developmental programs, whose manipulation can alter stem cell properties and lead to cancer [12]. Multiple cellular pathways induce and maintain the specific stemness of stem cells, among which the regulation of the ubiquitin–proteasome system plays a significant role [13]. In this article, we review the importance and effect of transcription factors, signaling pathways, and cellular microenvironments for the survival, apoptosis, and metastasis of CSCs. We also describe the role of deubiquitinating enzymes (DUBs) in CSC-related signaling pathways and their implications for CSC therapy. 

### 1.1. Ubiquitination and Deubiquitination

**Figure 1:**
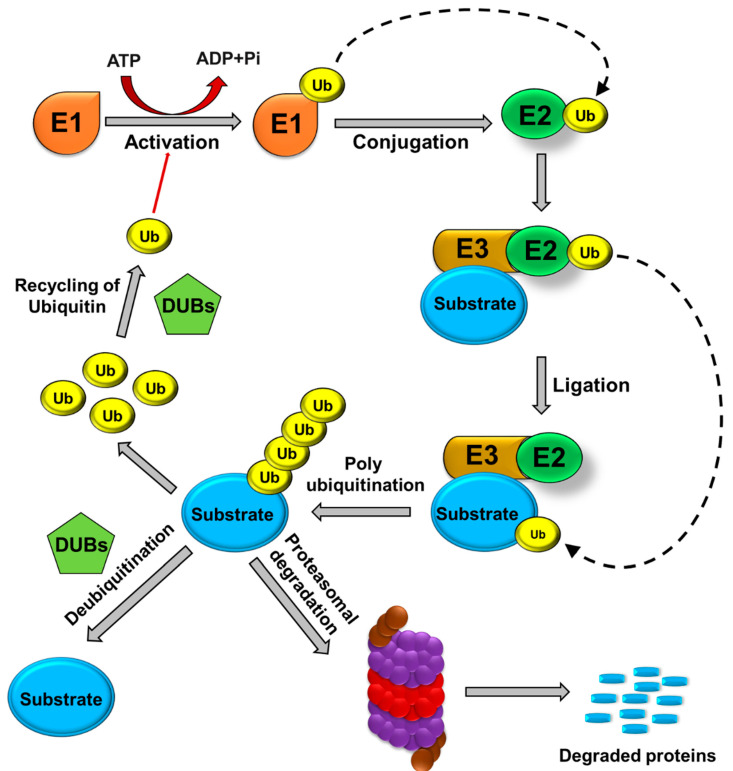
Ubiquitin proteasomal pathway. E1 (ubiquitin-activating enzymes), E2 (ubiquitin-conjugating enzymes), and E3 (ubiquitin ligases) are involved in the binding of ubiquitin molecules to protein substrates. Polyubiquitinated protein substrates are targeted to the 26S proteasome for proteolysis. Ubiquitin molecules are recycled, and protein degradation is prevented by the action of DUBs.

Ubiquitination is a post-translational modification (PTM) process by which the highly conserved 76-amino acid ubiquitin protein is covalently conjugated to a lysine residue on a substrate protein as the result of a cascade of enzymatic reactions [14]. The first enzyme, E1 (Ubiquitin (Ub) activating), forms a covalent intermediate with ubiquitin, driven by the ATP-dependent activation of the C-terminus of ubiquitin. The terminal glycine of ubiquitin is linked to a cysteine residue’s thiol group in the E1 active site. The activated ubiquitin is transferred to the cysteine residue of the second enzyme in the cascade, E2 (Ub conjugating), where it forms a thioester-linked E2-Ub intermediate. Finally, the third enzyme, E3 (Ub ligase), catalyzes the transfer of ubiquitin to a lysine residue in the target protein substrate and forms an amide bond [15]. The target protein can be ubiquitinated at single or at multiple lysine residues through addition of ubiquitin moieties as monomers or polymers (polyUb) at various topologies [15] (Figure 1). Topology indicates the spatial arrangement of Ub subunits and is regulated by the links among ubiquitin subunits [16]. Both monomers and polymers control the fates of the proteins to which they become covalently attached. 

Monoubiquitination, the attachment of short Lysine (K)63 -linked ubiquitin chains to a protein, has primarily been linked to chromatin regulation, protein sorting, and trafficking. In contrast, polyubiquitination has chains of more than three ubiquitins, with the C-terminus of one ubiquitin connected to the K48 of the following ubiquitin, and it has been associated with protein signaling and clearance through proteasomal degradation [17,18]. The cellular mechanisms made possible by ubiquitin signaling are driven by polyUb, which uses both homotypic (same positional linkages) and heterotypic (linkages at multiple sites) chain topologies and linkages [19]. PTM of polyUb can produce several topologies that affect different functions within the cell. Any modification in these chains can mediate their biological activity and confound their characterization [19]. Crosstalk between ubiquitination and other PTMs uses bidirectional regulatory mechanisms. Any modification in the Ub and its receptors through acetylation, phosphorylation, or deamidation can diversify the Ub code and have important therapeutic implications. Recent work on the PTEN-induced kinase 1 (PINK1)/Parkin ubiquitin ligase discusses crosstalk between ubiquitination and phosphorylation in the selective mitophagy pathway, wherein the PINK1 kinase activates Parkin by phosphorylating the Ubl domain of Parkin and the Serine 65 of Ub, promoting the autophagy signal needed to remove damaged mitochondria [20].

**Figure 2:**
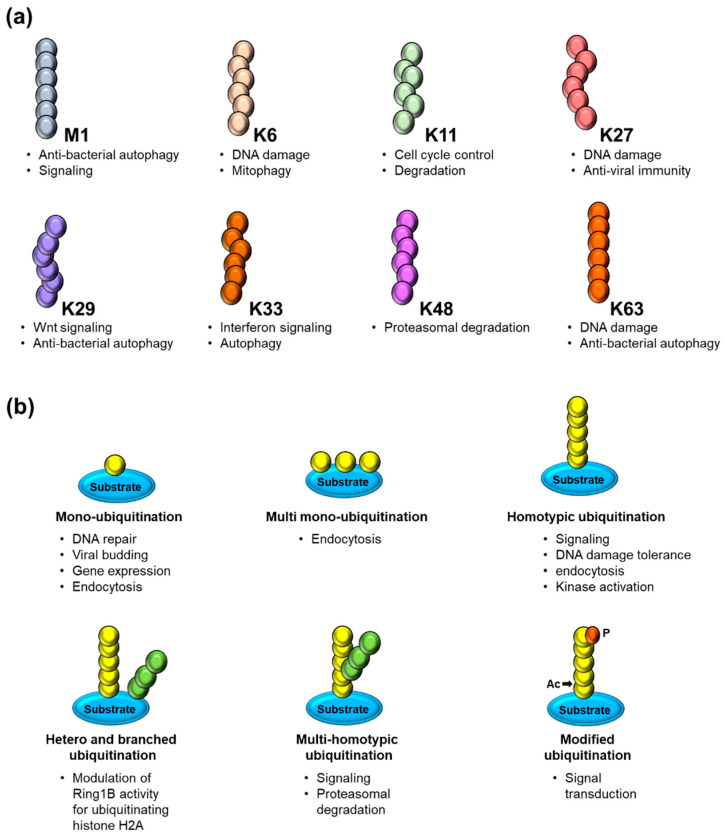
(**a**) Schematic representation of ubiquitin chains linked through methionine (M) 1 (linear/head–to–tail) or through the internal lysine (K) residues 6, 11, 27, 29, 33, 48 and 63 along with its respective cellular functions. (**b**) Overview of several modes of substrate ubiquitination including different forms of mono– and polyubiquitination and the PTMs of ubiquitin itself by acetylation (Ac) and phosphorylation (P) along with its respective cellular functions.

Ubiquitin has seven Lys residues that can be ubiquitinated to form isopeptide-linked ubiquitin chains. Proteomics studies have revealed the existence of all possible linkage types in cells [21]. Various ubiquitin modifications linked through Methionine1, K6, K11, K27, K29, K33, K48, and K63 have linkage-specific enzymes and proteins that assemble, recognize, and hydrolyze each ubiquitin chain type (Figure 2). Among them, the K48-linked chains are predominant and target proteins to proteasomal complexes for degradation [21]; K63 has a role in promoting non-degradation in inflammatory signaling and DNA repair [21]; K11-linked chains regulate endoplasmic reticulum-mediated degradation and cell cycle progression [22]. K33 is involved in intracellular trafficking [23], and K29-linked chains influence proteasome regulation and epigenetics [24]. The K6 polyubiquitin chain is involved in auto ubiquitination of the breast and ovarian cancer suppressor BRCA1 and the DNA repair process [25]; the K27 residue (UbK27) targets histones H2A/H2AX and is required for RNF168-dependent chromatin ubiquitination during the stress response [26]. Ubiquitination also plays a role in various biological events, such as apoptosis, cell cycle control, oncogenesis, immune responses, embryonic development, transcriptional regulation, and intracellular signaling pathways [27].

The ubiquitination process is reversed by specialized enzymes—i.e., DUBs, that oppose the action of the E3 ligases by cleaving the iso-peptide linkage between the N-terminal lysine and the C-terminal glycine ubiquitin residues. Analyses of the human genome have identified about 100 functional DUBs that have been divided into seven broad classes based on active site homology: ubiquitin-specific proteases (USPs), ubiquitin carboxy-terminal hydrolases, ovarian-tumor proteases, Machado–Joseph disease protein domain proteases, JAB1/ Mpr1, Pad1 N-termina (MPN)/Mov34 metalloenzyme (JAMM/MPN domain-associated metallopeptidases, and monocyte chemotactic protein-induced protein (MCPIP) [28,29]. More recently, additional DUB subfamily members have been identified, including MINDY (motif interacting with Ub-containing novel DUB family) and ZUFSPs (Zn-finger and UFSP) domain proteins [30,31]. The deubiquitination of proteins is highly specific and essential for regulating biological processes, such as transcription, PTMs, substrate activation, and rapid degradation [32]. Deubiquitination maintains a steady level of monoubiquitin and regulates proteasomal substrate degradation, histone modification through chromatin remodeling, the cell cycle, the DNA damage repair mechanism, endocytosis, and activation of several kinases and enzymes [27,33]. Among the DUB sub-families, the USPs are highly diversified and comprise more than 50 members, forming the largest sub-family of DUBs. USPs undergo mutations in multiple biological processes and are frequently altered in CSCs. Thus, the altered expression of USPs has a relationship with tumor progression; however, the roles played by many USPs in cancer and CSC biology remain unexplored [28]. 

### 1.2. DUBs and Stem Cell Fate Determinants

**Figure 3:**
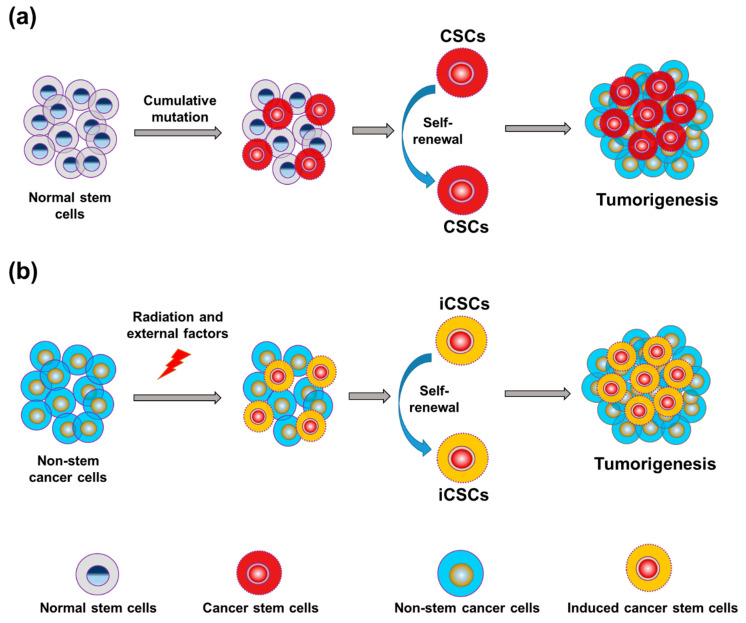
Progression of tumors from normal stem cells and non-stem cancer cells. (**a**) Differentiation of cancer stem cells (CSCs) within the pool of stem cells is a major source of tumorigenesis. (**b**) Radiation and other external factors can form induced cancer stem cells (iCSCs) in the pool of non-stem cancer cells, resulting in tumorigenesis.

The metastatic potential of tumors is determined by the prevailing lineage-specific cell fate determinants, which can include transcriptional regulators expressed in niche cells. Any modification or loss of controlled differentiation can result in the development of metastatic characteristics, such as de-differentiation, procurement of stem cell-like activities, or cellular plasticity [34]. Multiple cell types, such as healthy stem cells, directed group progenitor cells, mature cells, and a fusion of stem cells and other mutant cells, can give rise to CSCs (Figure 3). The transformation of normal cells into CSCs results from multiple gene mutations, epigenetic changes, uncontrolled signaling pathways, or dysregulation of the microenvironment vital for cellular survival and growth. Both CSCs and embryonic stem (ES) cells can grow indefinitely and self-renew, and they share many signaling pathways and some transcription factors. However, it is not known whether embryonic transcription factors can become re-expressed or reactivated in CSCs. 

Somatic cells can be reprogrammed to become pluripotent stem cells through transient ectopic overexpression of transcription factors that play an essential role in regulating the growth of CSCs, such as Oct3/4, Sox2, Nanog, KLF4, c-Myc and other reprogramming factors [35]. These factors have been found in multiple cancers, such as breast, lung, bladder, and head and neck [36]. Recent studies have revealed the importance of ubiquitin modification to stem cell factors, ESC regulators, and differentiation-related factors [37,38]. Those findings suggest that ubiquitination and deubiquitination regulate stem cell transcription factors and influence the efficiency of cellular reprogramming.

Octamer-binding transcription factor 4 (Oct4), a homeodomain transcription factor of the Pit-Oct-Unc family, is recognized as one of the most important transcription factors [39]. Oct4 is highly expressed in CSCs, is positively correlated with gliomas, and promotes self-renewal, chemoresistance, and tumorigenicity in Hepatocellular carcinoma (HCC) stem cells [40]. Ubiquitination of Oct3/4 is regulated by the E3 ligase Wwp2 in mouse cells (WWP2 in human cells), which mediates degradation of the Oct3/4 protein by attaching Lys−48- or Lys−63-linked polyubiquitin chains [37,41]. Additionally, Psmd14 interacts with the lid of the 19S proteasome, altering its on/off function. Upon depletion of Psmd14, the activation of the proteasomal lid is disrupted, ultimately leading to the accumulation of both K48- and K63-linked polyubiquitinated proteins. That leads to a significant decrease in Oct4 protein expression and abnormal ESC morphology [42]. Therefore, Oct4 is a pluripotent factor in CSCs, and PTMs play a significant role in its processes.

SRY (sex determining region Y)-box 2 (Sox2) is a crucial transcription factor for CSCs and regulates their differentiation and stemness. Sox2 belongs to a family of high-mobility group transcription factors and plays a significant role in early development and maintenance of undifferentiated ESCs [43]. Phosphorylation of Sox2 protein by protein kinase B (PKB), also known as Akt stabilizes Sox2 by antagonizing protein degradation, whereas Set7 monomethylates Sox2 and recruits the additional E3 ligase WW domain-containing protein 2 (WWP2), which inhibits Sox2 ubiquitination and degradation [38]. With regard to transcriptional regulation of human ESCs, numerous DUBs have been studied based on genome-scale observations. Sox2 associated with USP9X regulates the growth of tumor cells in the brain [44]. In contrast, rather than controlling the stability of targeted proteins, several DUBs can act as transcriptional repressors in ESCs and are required for efficient differentiation. DUBs can bind to the promoter region of Sox2, hydrolyzing monoubiquitin from the ubiquitinated H2B (uH2B) that blocks its transcription. Sox2 is negatively regulated by USP22, which is present on the promoter of the Sox2 gene in ESCs [45]. Consistent with its effect on stemness of CSCs, Sox2 can regulate the activity of DUBs, such as USP7, USP25, USP37, USP44, and USP49 at the transcriptional level [46]. Those studies indicate the importance of Sox2 ubiquitination and deubiquitination.

Krüppel-like factor 4 (KLF4) is expressed in many tissues, plays an essential role in many physiological processes, and acts as a bifunctional transcription factor (activator or repressor depending on the function of the target gene). Ubiquitination of KLF4 is a vital PTM that can control its turnover within cells. In response to TGF-β signaling, KLF4 expression is downregulated and inhibited by the ubiquitin–proteasome pathway [47]. Kim et al., in 2012, showed that ERK-mediated phosphorylation of KlF4 induces βTrCP1 or βTrCP2 binding to the N-terminal domain of Klf4. βTrCP1 and βTrCP2, which are components of the ubiquitin E3 ligase, signal Klf4 ubiquitination and degradation [48]. The Cdh1/ Anaphase promoting complex (APC) E3 ubiquitin ligase also interacts with and regulates TGF-β-induced KLF4 proteolysis [48]. In ESCs, USP9X depletion unbalances the pluripotency transcription factor LEFTY2 (a regulator of TGF-β signaling) and pluripotency factors ESRRB and KLF4 [49]. Additionally, Wang et al. recently reported that the depletion of USP10 promotes KLF4 degradation and enhances the progression of lung adenocarcinoma [50]. Another DUB, MCPIP, is involved in treatment with murine peritoneal macrophages [51]. These studies suggest the role of E3 ligases and other PTM activities in regulating KLF4 function.

Myc is a family of proto-oncogenes that encode transcription factors critical to DNA binding proteins of the basic helix–loop–helix (bHLH) superfamily [52]. Myc controls protein-coding and non-coding genes that coordinate multiple biological processes, such as cell growth, differentiation, metabolism, self-renewal, and development of stem cells [53]. PTMs regulate the function and stability of c-Myc through ubiquitination and proteolysis. The c-Myc proto-oncogene is a classical CSC-related marker that is stabilized by many DUBs. USP22, an enzymatic subunit of the human Spt-Ada-Gcn5 acetyltransferase (hSAGA) transcriptional cofactor complex, is recruited by Myc to specifically target gene transcription and produce angiogenesis, growth, and metastasis in non-small cell lung cancer [54]. USP28 stabilizes c-Myc in human tumor cells, where it binds to c-Myc by interacting with FBW7 alpha, an F-box protein that is part of an SCF-type ubiquitin ligase [55]. USP28 positively regulates c-Myc stability and tumorigenic activity in normal mammalian and breast cancer cells. USP37 directly deubiquitinates and stabilizes c-Myc in lung cancer [56]. In a subset of human breast and lung cancers, USP36 interacted with and deubiquitinated c-Myc [57]. These studies indicate that c-Myc has various activities in CSCs, and that the ubiquitination and deubiquitination of c-Myc critically regulate cellular functions.

Nanog, a differentiated homeobox (HOX) domain protein, has multipotent transcriptional regulatory function and self-renewal capability [58]. Nanog is downregulated in normal somatic cells; however, it is expressed anomalously in numerous cancers, including brain, breast, colon, head and neck, lung, and cervical [58,59,60,61]. In colorectal CSCs, Nanog is overexpressed and enhances colony formation and tumorigenicity in vivo [62]. Nanog is highly expressed in gastric cancer, where it is associated with poor patient survival [60], and in HCC cell lines, where it is linked with advanced disease [63]. Nanog is an essential stemness marker that is regulated by PTMs. USP7 binds directly with the BTB and CNC homology 1 (Bach1) transcription factor, which interacts with Nanog, Sox2, and Oct4 in human ESCs [64], and USP21 maintains the stemness of mouse ESCs by stabilizing Nanog through the removal of its K48-linked ubiquitin chains. It also deubiquitinates and stabilizes Nanog to facilitate target gene expression [65,66,67,68]. Phosphorylation of the Nanog protein by ERK1 decreases the stability of Nanog through increased binding and the ubiquitination of the E3 ligase FBXW8 [69]. These studies indicate that the PTM of Nanog regulates CSC self-renewal and proliferation. 

Inhibitors of DNA binding (ID) proteins are transcriptional regulators that control cell fate determination and the timing of differentiation in progenitor stem cells during normal development [70]. ID proteins are one of the molecular targets for the treatment of breast and other cancers [70,71]. USP1 deubiquitinates and stabilizes ID1, ID2, and ID3 proteins in osteosarcoma [72]. USP1 inhibition promotes ID1 degradation and is cytotoxic to leukemic cells. Additionally, the USP1/WDR48 complex regulates cell proliferation and differentiation in CSCs through ID proteins [73]. DUBs associated with stem cell factors are summarized in Table 1.

## 2. DUBs Involved in Stemness-signaling Pathways Related to CSCs

This section will cover signaling pathways, such as Sonic hedgehog, Wnt, Notch, TGF-β and Hippo, and others that regulate CSC properties. 

### 2.1. Hedgehog Pathway

The Hedgehog (Hh) pathway plays a significant role in the development and patterning of numerous organs during embryogenesis by influencing cellular differentiation, proliferation, and migration. The Hedgehog pathway includes three secreted Hedgehog ligands—Sonic, Desert, and Indian—their cognate receptor Patched, the transmembrane protein Smoothened, and three Gli translation components that balance the activation and suppression of the pathway. Hh activity regulates normal stem cell (NSC) populations, and aberrant Hh signaling is associated with many cancers, including colon cancer, basal cell carcinoma, glioblastoma, multiple myeloma (MM), adenocarcinoma, bladder cancer, esophageal squamous cell carcinoma, gastric cancer, lung squamous cell carcinoma, and chronic myeloma leukemia (CML) [74]. The Hh pathway is responsible for maintaining cell polarity and stemness by driving the expression of various stemness-related genes, including Sox2, Oct4, and Bmi1 [75,76,77,78]. Treating glioma CSCs with an Hh signaling inhibitor reduced multiplication, survival, self-renewal, and clonogenicity of the CSCs and diminished expression of stemness genes, such as Nanog, Sox2, and Oct4 [79]. 

Gli proteins are transcriptional effectors of Hh signaling; several DUBs are reported to interact and stabilize Gli proteins. For instance, USP7 acts as a positive regulator of Hh signaling by deubiquitinating and stabilizing Ci and Gli proteins, indicating that Gli is a significant potential therapeutic target for Hh-related cancers [80]. Another DUB, USP21, is centrosome-associated, helps in the formation of primary cilia-crucial organelles for regulation of the Hh signaling pathway―it interacts with Gli, and suppresses Gli-dependent transcription [81]. USP48 interacts and deubiquitinates Gli1 proteins by cleaving off their ubiquitin molecules to enhance their half-life. There is a reciprocal feedback loop between the expression of USP48 and Gli by which USP48 stabilizes Gli1 proteins and Gli1 activates USP48 transcription. This crosstalk between USP48 and Gli1 leads to sustained activation of the Hh signaling pathway, which promotes cell proliferation, tumor progression, and CSC self-renewal [82].

The expression of USP37 is critical in the maintenance of key Hh components. Genetic depletion of USP37 leads to a reduction in the protein level of key Hh components and stem cell markers. In contrast, the activation of Hh signaling by purmorphamine results in the up-regulation of USP37, which, in turn, leads to the stabilization of Gli1 and impacts on Epithelial–mesenchymal transition (EMT) in breast cancer stem cells [83].

Hh signaling is critical to osteoblast differentiation during embryogenesis. OTUB2 deubiquitinates and stabilizes the GLI2 protein by preventing its proteasomal degradation. Moreover, depletion of OTUB2 reduces the expression level of Hh target genes, such as N-myc, Patch1, and Gli1, leading to the suppression of Hh-induced osteoblast differentiation in mesenchymal stem cells (MSCs) [84]. 

USP8 promotes Hh signaling by preventing its ubiquitination and changing its subcellular localization, where it removes modified ubiquitin from Smo that enhances its signaling activity [85]. Usp14 plays a major role in ciliogenesis, ciliary length and Hh pathway activation. Overlapping phenotypes of ciliopathies are associated with mutations in genes coding for proteins localized to the primary cilia and regulate cilia elongation and functionality. Pharmacological inhibition of Usp14 positively affects Hh signal transduction in the model of autosomal dominant polycystic kidney disease [86].

### 2.2. Wnt/β-catenin

The Wnt/β-catenin signaling pathway plays an essential role in tissue self-renewal and cell fate determination and is involved in tumorigenesis [87]. The Wnt signaling pathway is evolutionarily conserved and profoundly complex, including more than a dozen Wnt ligands and their receptors [88]. Generally, the Wnt pathway comprises canonical (β-catenin-dependent) and non-canonical (β-catenin-independent) signaling. The canonical pathway is activated upon binding between secreted Wnt ligands (for example, Wnt3a and Wnt1) and Frizzled (FZD) receptors and Lipoprotein receptor-related protein (LRP) co-receptors. The LRP co-receptors are phosphorylated by CK1α and GSK3β to recruit dishevelled (Dvl) proteins to the plasma membrane where they polymerize and are activated. In the non-canonical pathway, the Wnt ligand that reduces the action of the destruction complex (Axin, adenomatous polyposis coli (APC) kinase proteins, glycogen synthase kinase−3b (GSK−3b) and casein kinase 1a (CK1α)) is absent. GSK−3b and CK1α phosphorylate β-catenin, labeling it for ubiquitination and consequent proteasomal degradation. Additionally, when a Wnt ligand binds to (FZD)/LRP receptors, the cytoplasmic domain of LRP is phosphorylated and sequesters GSK−3b and Axin to ultimately recruit Dvl. This dismantles the destruction complex such that the free β-catenin translocates into the nucleus and binds to lymphoid enhancer factor (LEF) or T-cell factor (TCF) transcription factors, activating transcription of various target genes, such as Wnt/Wg and Myc [89].

Recent research has focused on the role of Wnt activation in regulating CSCs in cancers of the mammary glands, skin, nervous system, intestines, lungs, urinary tract, and blood [90]. Loss of the tumor suppressor APC gene tends to result in familial and sporadic colorectal cancer (CRC) in humans [91]. Wnt signaling plays an essential role in both development and tumorigenesis. To maintain proper cell activity, a balance between Wnt ubiquitylation and deubiquitylation and its receptor FZD is critical. USP2a interacts with, stabilizes, and deubiquitinates β-catenin and promotes nuclear accumulation and transcriptional activity of β-catenin to upregulate Wnt/β-catenin target gene expression. Inhibiting USP2a with ML364, a small-molecule inhibitor, downregulates β-catenin in cancer cells, suggesting that USP2 might be a therapeutic target to reduce the cancer-promoting protein β-catenin [92]. USP15 prevents proteasomal degradation of β-catenin, which reduces its basal turnover and enhances Wnt signaling. USP15 was recruited by FGF2-mediated phosphorylation of β-catenin [93] and is involved in the stability of the COP9 signalosome, Cullin-RING ubiquitin ligases, and β-catenin destruction complex through stabilization of adenomatous polyposis coli (APC), indicating that it affects Wnt/β-catenin signaling [94].

USP4 positively regulates Wnt/β-catenin signaling in colon cancer. The C-terminal catalytic domain of USP4 is responsible for β-catenin binding and nuclear transport. Knockdown of USP4 in a colon cancer cell line decreased invasion and migration activity, indicating that USP4 is a positive regulator of β-catenin and is a potential target for anti-cancer therapeutics [95].

USP6 activates Wnt signaling by deubiquitinating FZD and increasing its cell surface abundance, which is an important mechanism in regulating sensitivity to Wnt ligands. Chromosomal translocations in nodular fasciitis promote USP6 overexpression, which results in transcriptional activation of the Wnt/β-catenin pathway. During tumorigenesis, Wnt signaling is the main target of USP6 because inhibition of Wnt signaling decreases USP6-driven xenograft tumors [96]. Ubiquitin-specific protease 6 N-terminal-like protein (USP6NL) or Tre2, a homolog of USP6, has a domain that encodes a DUB enzyme. Knockdown of USP6NL increased the ubiquitination of β-catenin, suggesting that it could act as a DUB that regulates β-catenin accumulation. Downregulation of USP6NL suppressed cell proliferation and other β-catenin-targeted genes involved in transition from G0/G1 to S phase. In CRC patients, the expression level of USP6NL was higher in tumor tissues than in normal tissues. Thus, USP6NL behaves as an oncogene and can be a potential therapeutic target for CRC [97]. USP8 deubiquitinates endosome-tethered FZD protein, which recycles FZD to the plasma membrane by regulating ubiquitination, lysosomal trafficking, and degradation. Inhibition of USP8 reduces cell-surface FZDand suppresses the cellular response to Wnt in the canonical pathway [98]. 

USP9X stabilizes and deubiquitinates β-catenin by removing the Lys 48-linked polyubiquitin chains that mark it for proteasomal degradation, thereby promoting glioma cell proliferation and survival and indicating it as a therapeutic target in high-grade gliomas [99]. Additionally, USP9X-mediated B-cell CLL/lymphoma 9 (BCL9) deubiquitination facilitates formation of the β-catenin-BCL9-PYGO complex, which increases the transcriptional activity of the Wnt/β-catenin target genes. USP9X binds with BCL9 and removes the Lys−63-linked polyubiquitin molecules. Deubiquitination of BCL9 by USP9X also promotes the proliferation and invasion of breast cancer cells, implying the role of USP9X in breast carcinogenesis [100].

Usp14 deubiquitinates Dvl, a key regulator of Wnt signaling. Suppression of Usp14 activity leads to increased Dvl polyubiquitination and impairs downstream factors of Wnt signaling, suggesting that Usp14 is a positive regulator of the Wnt signaling pathway [101]. USP14 inhibition combined with the drug enzalutamide enhanced the sensitivity of breast cancer compared with single-drug treatment [102]. Cylindromatosis (CYLD) also functions as a negative regulator of Wnt signaling by deubiquitinating the cytoplasmic effector Dvl. CYLD silencing enhances Wnt-induced accumulation of β-catenin and target gene activation [103].

USP25 acts as a positive regulator of Wnt/β-catenin signaling. USP25 interacts with and deubiquitinates tankyrase, which destabilizes Axin and positively regulates Wnt signaling [104]. USP47 also acts as a positive regulator of Wnt/β-catenin signaling by stabilizing β-catenin through its deubiquitinating activity [105]. Another family of DUBs, TRABID, positively regulates Wnt signaling by deubiquitinating APC, which regulates the stability of β-catenin [106].

In contrast, several DUBs act as negative regulators of Wnt/β-catenin signaling. For instance, USP7 inhibits Wnt/β-catenin signaling by promoting stabilization of Axin by direct interaction through its N-terminal TRAF domain and stabilizes and deubiquitinates the Axin protein. Depletion of USP7 promotes osteoblast and adipocyte differentiation by activating Wnt signaling. Thus, USP7 is a specific target for sensitizing cells in response to Wnt/β-catenin signaling [107]. USP34 stabilizes Axin and opposes tankyrase-dependent ubiquitination of Axin, indicating that USP34 negatively regulates Wnt/β-catenin signaling [108]. Similarly, USP44 acts as a negative regulator of Wnt/β-catenin signaling by deubiquitinating and stabilizing Axin. The increased expression of USP44 upregulates the expression of Axin but downregulates β-catenin, c-Myc, and cyclin D1 expression [109].

### 2.3. Notch Pathway

The Notch pathway consists of a cascade of signaling molecules involved in development of cells and maintenance of stemness [110]. Notch signaling is activated when a ligand delta-like protein precursor (DLL) (DLL1, DLL3, DDL4)Jagged1, or Jagged2) expressed on one cell communicates with any of its corresponding receptors (Notch 1–4) on an adjacent cell [111]. This binding initiates proteolytic cleavage of the cytoplasmic domain of the receptors by releasing disintegrin, metalloproteinases and g-secretase. This cleavage discharges the Notch intracellular domain (NICD) into the cytoplasm, followed by translocation into the nucleus to activate target gene transcription [110,111].

Notch signaling denotes a primordial, cell-fate-determining, and evolutionarily conserved pathway with strong relevance to CSCs. Various studies have revealed that the Notch pathway is involved in regulation of CSCs in pancreatic cancer, esophageal adenocarcinoma, breast carcinomas, and many other cancers [112,113,114].

Notch signaling plays a role in regulating neural stem cell expansion in vivo and in vitro [115]. In vivo studies have shown that DUBs, such as BAP1, eIF3F, eIF3H, and USP10 are responsible for regulating the Notch pathway in mammary cells [116].

USP7 acts as a regulator of UbE2E1, an E2 ubiquitin conjugation enzyme with a unique N-terminal extension. USP7 attenuates UbE2E1-mediated ubiquitination, an effect that requires the N-terminal ASTS sequence of UbE2E1 as well as the catalytic activity of USP7 [117]. Another report suggested that USP7 blocks Itch-mediated degradation of Gli1 and Spop-Cul3-mediated degradation of Gli2/Gli3 without affecting Gli mRNA levels [80]. USP7 also deubiquitinates and stabilizes Notch1 in acute T-cell lymphoblastic leukemia [118]. 

USP9X interacts with and stabilizes ITCH, an E3 ligase that ubiquitinates Notch. USP9X interacts with and protects Itch auto-ubiquitination through its deubiquitinating activity [119]. USP12 acts as a negative regulator of the Notch pathway by reversing ITCH-mediated ubiquitination of the Notch ligand. USP12 silencing interrupts Notch trafficking to the lysosomes by increasing the quantity of surface-expressed Notch receptors, increasing Notch activity [120]. 

USP9X deubiquitinates Mind Bomb (MIB) 1, an E3 ligase required for Jagged1 ubiquitination-mediated endocytosis and Notch activation [121]. Tribbles homolog 3 (TRB3) regulates JAG1 expression during malignancy and is involved in Notch signaling [121]. Upon cellular stress in basal-like breast cancer, USP9X is required for TRB3 upregulation and Notch activation. Thus, TRB3 acts as a sensor during tumor environmental stress and functions with USP9X to induce cell survival and tumor-promoting activities of Notch. CYLD stabilizes MIB2 and reduces the level of JAG2, a ligand implicated in Notch signaling activity. Notch pathway activity in skin tumors from patients with germline mutations in *CYLD* showed that the JAG2 protein levels and Notch target genes were upregulated [122].

USP10 stimulates Notch signaling in the endothelium by binding with the Notch1 intracellular domain (NICD1). USP10 stabilizes NICD1 by extending its half-life, which is necessary for prolonged cellular Notch responses. Inactivation of USP10 reduces NICD1 and subsequently downregulates Notch-induced target gene expression in endothelial cells, indicating its importance in endothelial Notch responses during angiogenic sprouting [123]. 

USP11 deubiquitinates and stabilizes promyelocytic leukaemia (PML) to control Notch-induced malignancy in brain tumors. The Notch effector Hey1 is recruited to the USP11 promoter to repress expression of USP11. Thus, Notch-induced downregulation of USP11 and PML promotes multiple malignant features of glioblastoma multiforme (GBM) and glioma-initiating cells (GICs), indicating the importance of this pathway in GBM malignancy [124]. 

### 2.4. TGF-β/BMP Signaling 

The transforming growth factor-β/bone morphogenic proteins (TGFβ/BMP) signaling pathway controls several processes, such as cell differentiation, proliferation, survival, and motility of cells [125]. The TGF-β ligands (TGF-β/activin and BMP/GDF) bind to the Type II TGF-β receptor, which recruits the Type I TGF-β receptor and phosphorylates R-Smads, leading to regulation of gene expression [126]. Dysregulation of any of these signaling processes can lead to malignant transformations and cancer stemness [127]. Other CSCs regulated by this pathway include those found in HCC; squamous cell carcinoma; glioma; liver, lung, breast, and gastric cancers [128]. 

USP2a positively regulates the TGF-β signaling pathway by removing the K33-linked ubiquitin chain. USP2a binds with heterodimeric type II and type I TGF-β serine/threonine kinase receptors (TGFBR1 and TGFBR2) upon TGF-β stimulation and promotes the recruitment of SMAD2/3. The phosphate groups on the Ser207/Ser225 of USP2a are phosphorylated by TGFBR2, which disassociates SMAD2/3 from TGFBR1. The phosphorylation of USP2a and SMAD2 is positively linked in tumor tissues, with USP2a hyper-phosphorylated in lung cancers. The inhibition of USP2a impairs TGF-β-induced EMT and metastasis, suggesting USP2a as a potential target for the treatment of metastatic cancers [129].

USP4 directly interacts with and deubiquitinates type 1 TGF-β receptors that control TβRI level at the plasma membrane. Akt, a serine/threonine-specific protein kinase, phosphorylates USP4, which promotes its re-localization from the nucleus to the membrane, where it binds with TβRI. The depletion of USP4 restricts TGF-β-induced EMT and metastasis [130].

USP9X enhances TGF-β signaling by countering SMAD4 monoubiquitylation. USP9X binds and deubiquitinates monoubiquitinated Smad4, opposing the activity of Ectodermin, which reversibly represses Smad4 activity [131]. The depletion of USP9X disorganizes the developing neocortex architecture in early neural progenitor populations and the axonal projections of the neurons of the cortical plate. The loss of USP9X also reduced the size of the hippocampus and axonal length, which suggests its importance in regulating TGF-β-signaling during central nervous system development [132].

USP11 binds and deubiquitinates Alk5, a type I TGF-β receptor, which results in enhanced TGF-β-induced gene transcription. USP11 knockdown mediated by *RNAi* inhibited TGF-β-induced phosphorylation of SMAD and TGF-β-mediated transcriptional responses [133]. USP11 targets both TGFBR1 and TGFBR2 for deubiquitination and enhances TGF-β signaling and metastasis, suggesting USP11 as a major therapeutic target for breast cancer [134,135].

USP15 enhances TGF-β signal by interacting with the SMAD7-SMAD E3 ligase (SMURF2) complex that is responsible for deubiquitination and stabilization of the type I TGF-β receptor. Moreover, high expression of USP15, which is predominantly expressed in glioblastoma and breast and ovarian cancer, correlates with high TGF-β activity [136]. UCH37 is associated with SMAD7 and influences TGF-β-mediated transcription during the early phase of TGF-β receptor activation. Additionally, UCH37 deubiquitinates and stabilizes the type I TGF-β receptor and upregulates TGF-β-dependent transcription [137]. USP26 acts as a negative regulator of the TGF-β pathway, which suggests that loss of USP26 expression correlates with high TGF-β activity and poor prognosis in glioblastoma. TGF-β enhances expression of USP26 and restricts ubiquitin-mediated degradation of SMAD7. Conversely, USP26 knockdown rapidly degrades SMAD7, resulting in TGF-β receptor stabilization and enhanced level of p-SMAD2 [138]. 

OTUB1 is critical for cellular migration and TGF-β-mediated gene transcription. Additionally, OTUB1 stabilizes the active SMAD2/3 complex by preventing the ubiquitination of phospho-SMAD2/3 through the inhibition of the E2 ubiquitin-conjugating enzymes, independent of its catalytic activity [139]. 

USP4 can suppress the TGF-β signaling pathway through a non-SMAD pathway. TNFα induces binding between USP4 and TAK1, resulting in deubiquitination of TAK1 and suppression of TAK1-mediated NF-κB activation [140]. CYLD acts as a negative regulator of TGF-β signaling by deubiquitinating Akt. CYLD destabilizes the Smad3 protein in a GSK3β-CHIP-dependent manner—the destabilization of Smad3 by CYLD is caused by direct deubiquitination of K63-linked polyubiquitinated Akt [141].

### 2.5. Hippo Pathway

The Hippo pathway is linked to regeneration and tumorigenesis [142]. Transcriptional regulators, such as yes-associated protein 1 (YAP) and the tafazzin (TAZ) transcriptional coactivator with a PDZ-binding motif, are involved in tumor initiation, self-renewal, and apoptotic resistance in CSCs, and they can reprogram tumors with differentiated profiles into CSCs with properties that confer resistance to anticancer treatments [142,143] The Hippo pathway plays a crucial role in preventing the activation of the YAP and TAZ regulators through large-tumor-suppressor−1 (LATS1)- and large-tumor-suppressor−2 (LATS2)-dependent phosphorylation, which repress the activity of these regulators and leads to increased cytoplasmic accumulation and stimulation of their ubiquitin-dependent protein degradation [142,144]. Accumulating evidence suggests that the Hippo pathway functionally interacts with several cellular pathways and acts as a central node in regulating cell multiplication, particularly in cancerous cells. This pathway also controls embryonic development and tissue homeostasis [145]. Sox2 antagonizes the Hippo pathway and maintains CSCs in osteosarcomas [146]. Hippo signaling is a critical regulator of several mammalian cancers, including liver, colon, breast, and ovarian [147]. The ubiquitin system regulates Hippo pathway activity, and the deregulation of ubiquitin ligases and DUBs increases YAP/TAZ activity in cancers [148]. The Hippo pathway is activated or repressed by the DUB enzymes summarized below.

USP9X influences the Hippo pathway by negatively regulating YAP/TAZ activity. The Angiomotin (AMOT) protein, which is involved in the repression of YAP/TAZ function, was deubiquitinated by USP9X at lysine 496 to stabilize AMOT and lower YAP/TAZ activity [149]. Additionally, USP9X stabilizes the LATS kinase, which suppresses YAP activity. Additionally, DUB3 stabilizes ITCH proteins, and its direct interaction with AMOT and LATS1/2 controls YAP turnover, DUB3 suppresses LATS phosphorylation and enhances nuclear localization of YAP [150]. USP47 interacts with and deubiquitinates YAP proteins that lead to the progression of CRCs. Both USP47 and YAP are highly expressed in CRCs, and their elevated expression is linked to CRC abundance [151].

USP11 deubiquitinates and stabilizes the vestigial-like family member 4 (VGLL4) protein, which directly competes with YAP by forming a complex with the TEA domain proteins (TEADs), thereby acting as a tumor suppressor. VGLL4 blocks the interaction of YAP and TEAD, reducing cell progression and migration. USP11 increases VGLL4 expression and modulates YAP-TEAD complexes by removing ubiquitin moieties from VGLL4 [152,153]. USP21 interacts with and stabilizes microtubule affinity-regulating kinase (MARK) proteins, which are upstream regulators of the Hippo pathway. The inhibition of USP21 suppresses LATS activity and increases nuclear YAP localization [154]. 

YOD1, a member of the ovarian tumor protease (OTU) subfamily of DUB enzymes, interacts with and regulates ITCH E3 ligases, resulting in the degradation of the LATS kinase that enhances the transcriptional activity of YAP and TAZ [155]. YOD1 is upregulated in 60% of human HCC as compared to normal tissues. Therefore, an increased level of YOD1 might play a tumorigenic role in liver cancer by suppressing Hippo activity, and its inhibitor could be used as a therapeutic tool to reactivate Hippo signaling in liver cancer [155]. 

OTUB2 stimulates CSC traits through angiogenesis. It interacts with and activates YAP/TAZ directly through sumoylation, which allows YAP/TAZ to interact with TEAD transcription factors and thus drives oncogenic properties both in vitro and in vivo [156]. OTUD1 regulates YAP in a Hippo-independent manner by reversing YAP’s non-proteolytic K63-linked polyubiquitination by the SCF-SKP2 E3 ligase complex that otherwise promotes its nuclear localization, transcriptional activity, and growth-promoting function with TEAD family transcription factors [157]. Moreover, Eukaryotic translation initiation factor 3 subunit H (EIF3H), a DUB and critical translational initiation factor, plays a major role in breast tumor invasion and metastasis by modulating the Hippo-YAP pathway. EIF3H stabilizes and deubiquitinates YAP to promote tumorigenesis [158].

**Figure 4:**
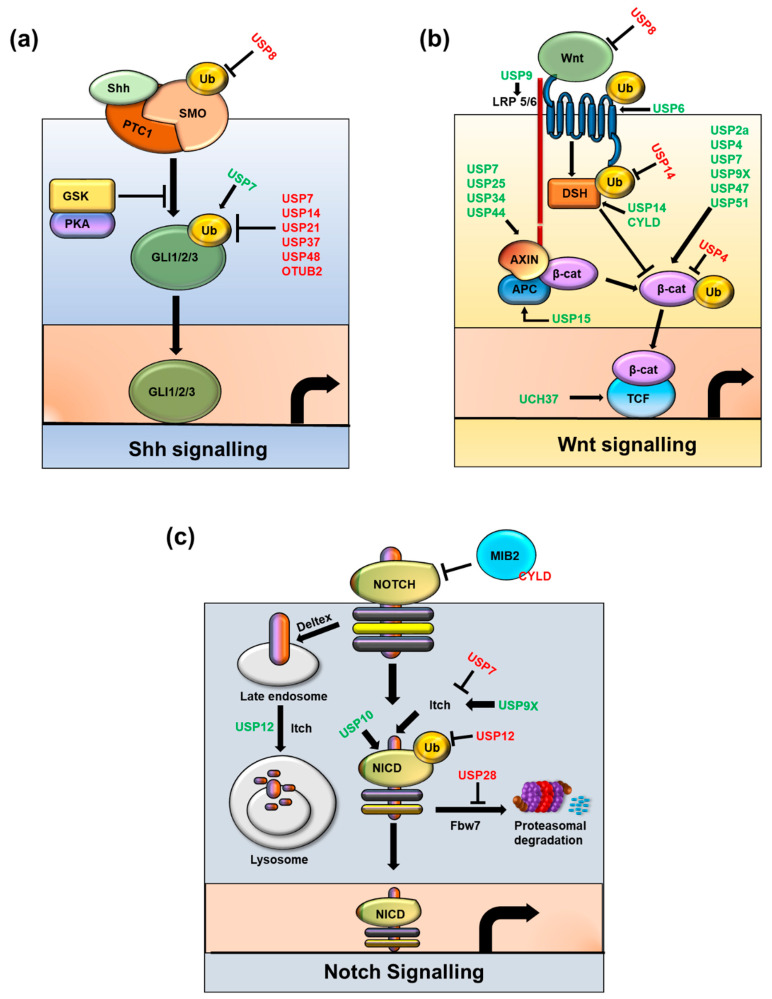

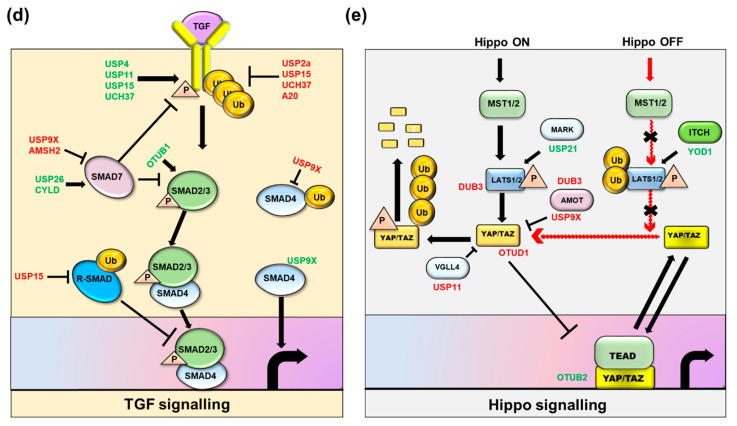
Regulation of Cancer stem cell-associated signaling pathways by key DUBs. Six main pathways that play significant roles in sustaining the stemness of Cancer stem cells are represented: (**a**) Sonic hedgehog signaling, (**b**) Wnt signaling, (**c**) Notch signaling, (**d**) Transforming growth factor signaling, and (**e**) Hippo signaling. Positive regulatory DUBs are depicted in green font and negative regulatory DUBs are depicted in red font.

Altogether, these findings demonstrate how the properties of the NSC signaling pathways, such as self-renewal, proliferation, differentiation, and survival, are unbalanced in tumorigenesis and CSCs, which are regulated by multiple exogenous/endogenous genes or microRNAs. These complex pathways are initiated by downstream signals, such as cytokines and developmental, proliferation, expression, apoptosis, anti-apoptotic, and metastatic genes in CSCs. The main DUBs which are discussed above, that play a critical role in regulating signaling pathways, such as Sonic hedgehog, Wnt, Notch, TGF-β and Hippo, are represented in Figure 4 and Table 2.

### 2.6. CSC Resistance-signaling Pathways

CSCs have much greater resistance to cytotoxic treatment than non-tumorigenic cells, indicating the importance of targeting CSCs to prevent tumor relapse. In CSCs, apoptotic proteins (Bim) are downregulated, and anti-apoptotic factors (Bcl−2, IAPs), DNA repair capacity, and caspase inhibitors are upregulated, contributing to therapeutic resistance [159]. Aldehyde dehydrogenase (ALDH) is a CSC marker that can help maintain a drug-tolerant subpopulation of cancer cells. Pharmacological disruption of ALDH activity leads to a toxic accumulation of reactive oxygen species (ROS), DNA damage, and apoptosis in the drug-tolerant subpopulation. [160]. The presence of ABC transporters in CSCs forms tumorospheres in non-adherent conditions, and their activity is elevated in xeno-transplantation studies [161,162]. Maintenance of the CSC population promotes cancer therapeutic resistance through crosstalk between signaling pathways, such as PI3K and Notch in breast cancer [163] and hedgehog and PI3K in biliary tract and pancreatic cancers [164]. Several DUBs can stabilize apoptotic and anti-apoptotic proteins. For example, USP9X stabilizes MCL−1 (BCL−2 family member) by removing polyubiquitin chains, protecting it from proteasomal degradation. Upon exposure to ionizing radiation, USP9X is activated and deubiquitinates Mcl−1, producing radioresistant activity in cells. This resistance behavior can be reversed by USP9X knockdown, which signals rapid degradation of Mcl−1 and induces apoptosis in radioresistant tumor cells [165].

The inhibitors of apoptosis (IAPs) are critical regulators of apoptosis and other fundamental cellular processes. The USP11-dependent mechanism selectively and specifically regulates cIAP2 stability independent of cIAP1 [166]. USP19 enhances TNFα-induced caspase activation and apoptosis in a c-IAP1- and 2-dependent manner [167], and OTUB1 disassembles K48-linked polyubiquitin chains from c-IAP1 within the (Tumor necrosis factor receptor superfamily member 12A) TWEAK receptor-signaling complex in vitro and in vivo. Downregulation of OTUB1 promotes TWEAK- and IAP antagonist-stimulated caspase activation and cell death and enhances c-IAP1 degradation [168].

Claspin is a checkpoint kinase 1 (Chk1) interacting protein that is essential for Chk1 phosphorylation and activation by the ATR kinase, which can also promote DNA replication fork progression and stability. Claspin and USP7 interact in vivo to maintain a steady-state level of Claspin [169]. HERC2/USP20, along with Chk1, modulates Claspin stability, which promotes genome stability and suppresses tumor growth [170]. Usp28 deubiquitinates and stabilizes Claspin upon DNA damage [171]. 

The anti-apoptotic protein FLIPS (caspase eight family inhibitors) is a crucial suppressor of tumor necrosis factor-related apoptosis-inducing ligand (TRAIL)–induced apoptosis. USP8, a downstream target of Akt, regulates the ability of Akt–atrophin-interacting protein 4 (AIP4) to control FLIPS stability and TRAIL sensitivity [172]. Chk1 is a master regulator of genome integrity in vertebrate cells that are regulated by DUBs. USP1 depletion stimulates the damage-specific DNA-binding protein 1-dependent degradation of phosphorylated Chk1 in both a monoubiquitinylated Fanconi anemia, complementation group D2 (FANCD2)-dependent and -independent manner [173]. USP3 regulates DNA damage by removing the K63-linked ubiquitin chain and releasing Chk1 from chromatin [174]. USP7 is a ubiquitin hydrolase for Chk1 whose inhibition decreases Chk1 protein level [175]. The Chk2-p53- (p53 mutant of apoptosis) PUMA axis regulates DNA-damage-induced apoptosis in response to double strand breaks. USP28 stabilizes Chk2 and 53BP1 in response to DNA damage by regulating p53 induction of proapoptotic genes, such as PUMA [176]. 

### 2.7. The CSC Microenvironment and Crosstalk between Signaling Pathways

Several ligands on one cell can activate corresponding receptors on neighboring cells, and those interactions can involve several cell types, as well as the extracellular matrix (ECM) and vasculature, in a dynamic microenvironment [177]. Such interactions among cells, their signaling pathways, and the surrounding microenvironment are thought to be critical to the maintenance of NSC and CSC populations [178,179,180,181]. For example, in colon CSCs, Wnt signaling is activated through myofibroblasts in the microenvironment in conjunction with inflammation [182]; in neural cancers, CSCs congregate in hypoxic areas of the tumor and perivascular niches [177]. In breast CSCs, several growth factors, ILs, chemokines, and other proteins secreted by fibroblasts, macrophages, endothelial cells, immune cells, and mesenchymal stem cells activate PI3K/PKB, STAT3, Wnt, and NF-kB signaling to form positive feedback loops between those and other cells in the microenvironment [183,184,185,186]. Cancer stemness can be enhanced by hyperactivation of these pathways, which are therefore potential targets for therapy. 

As noted above, the microenvironment of CSCs plays a significant role in maintaining stemness. To date, several signaling pathways in the CSC microenvironment—i.e., NF-κB, Hypoxia-inducible factors (HIFs), and Pattern recognition receptors (PRRs)—have been found to be associated with hypoxia, low ROS, hyper-angiogenesis, and immunosuppression [187,188,189,190]. Hypoxia is a critical factor in the tumor microenvironment and is a potential contributor to CSC phenotype. HIF−1α and HIF−2α are crucial transcription factors in the cancer hypoxia response and maintain several CSC populations [191,192]. In the presence of oxygen, VHL tumor suppressor proteins interact and degrade HIF proteins and maintain low levels of these mediators [193]. 

Crosstalk between signaling pathways is a crucial activity for maintaining the robustness of biological systems and can be initiated by a specific stimulus—i.e., an extracellular cue or individual gene suppressor or protein inhibitor. Several pathways act not just as a particular unit, but also as part of a biological network during cellular processes. Crosstalk among the Hh, Wnt, TGF, and other pathways has been reported in cancer and CSCs [194]. For instance, Hh signaling interacts with both Wnt and Notch signaling, where Wnt signaling is suppressed by Hh signaling in gastric cancer cells through soluble frizzled-related protein 1 (sFrP1). sFrP1 is a target gene of Hh signaling that regulates Wnt signaling by binding directly to the Wnt ligands and can serve as a molecular link for Hh signaling–mediated inhibition of Wnt signaling [195]. Wnt signaling also enhances the expression of Coding region determinant-binding protein (CRD-BP), an RNA-binding protein that interacts and stabilizes Gli1 mRNA, which in turn increases Gli1 transcriptional activity and promotes the survival and proliferation of CRCs [196]. In another study, Hh signaling was suppressed by Notch signaling through the hairy and enhancer of split−1 (Hes1) gene, a repressive transcription factor that binds with the first intron of Gli1 and inhibits its expression in glioblastoma-derived cells. The simultaneous inhibition of both Notch and Hh signaling hinders the growth of neurospheres, implying its therapeutic role in cancer treatment [197]. 

The interplay among complex signaling pathways is vital for maintaining embryogenesis, stem cell diversity, and tissue homeostasis. Dysregulation in these signaling networks leads to several pathological conditions, including cancer and other anomalies. TGF-β plays a significant role in EMT by activating the Smad pathway, in which lymphoid enhancer factor 1 (LEF1) is activated by phosphorylated Smad2 and binds with the promoter region of the E-cadherin gene to repress its transcription in response to the TGFβ3 signaling pathway [198]. Both LEF1- [199] and TGFβ3- [200] knockout mice have severe craniofacial deformities, including cleft palate. Additionally, it has been reported that TGF-β-induced EMT occurs through the NF-κB pathway in several cancers, where TGF-β activates the NF-κB through Long non-coding RNAs (LncRNA) NF-KappB Interacting LncRNA (NKILA) [201]. Moreover, TGF-β directly regulates the expression of Wnt5a in breast cancer, and Wnt5a acts as an effector of TGF-β actions in the branching of mammary gland development and cancer [202]. Crosstalk between TGF-β and the Hippo pathway has tumorigenic properties in osteosarcoma cells, where TAZ plays a functional role in modulating migration and cell proliferation in metastasis-derived cell lines [203]. The Hippo pathway also communicates with the Wnt signaling pathway and is involved in intestinal crypts. Hippo reduces the presence of free cytoplasmic β-catenin, which causes cell cycle arrest and affects crypt dynamics [204]. Thus, crosstalk among pathways by which they reinforce one another and regulate the self-renewal, survival, and metastasis of cancers can be seen in numerous tissues.

## 3. DUB Inhibitors in CSC-targeted Therapy

In cancer treatment, targeting tumorigenic CSCs directly is considered a more advanced technique than attempting to treat the tumor as a whole. Tumor recurrence after years of disease-free survival has incited interest in stem cells and intense investigation into the role of CSC self-renewal capacity and multi-lineage differentiation. DUBs protect the stemness of CSCs and regulate cell processes, such as cell proliferation, signal transduction, and apoptosis. DUB inhibitors target the most truculent DUBs, regulating CSC-related proteins and eliminating some of the challenges of cancer treatment, such as drug resistance and tumor recurrence. DUB inhibitors are superior to proteasomal inhibitors in curing refractory tumors—e.g., b-AP15, a DUB inhibitor targeting USP14 and UCHL5, can overcome resistance to bortezomib, a 20S proteasome inhibitor, in MM [205,206,207].

DUB inhibitors counteract the activity of DUBs associated with cancer stemness and EMT, including pimozide, which inhibits USP1 in osteosarcoma and glioblastoma [72,208]. ML323 is an inhibitor of USP1 that exhibits cisplatin cytotoxicity in non-small-cell lung carcinoma (NSCLC) and osteosarcoma cells [209].

HBX19818 is a potent inhibitor of USP7 that signals p53-mediated apoptosis in HCT116 colon cancer cells by blocking the deubiquitinating activity of USP7 [210,211]. P5091 is reported to target USP7 and USP47 and act as an anti-cancer agent in MM and HCT116 colon cancer cells [212,213]. MM cells generally show resistance to traditional chemotherapy agents, but P5091 induces their apoptosis by stabilizing p53 protein level and inhibiting cell proliferation [212,213].

P22077 and HBX19818, which was originally reported as an inhibitor of USP7, inhibit USP10 deubiquitinating activity, which produces anti-cancer activity [214]. Spautin−1 inhibits the USP10 and USP13 signals for rapid degradation of Beclin1 and Vps34 complexes that result in inhibition of autophagy [215]. Recently, spautin−1 was reported to trigger the apoptotic pathway in immunogenic cancer cells, resulting in cancer cell death in vivo and in vitro [216].

WP1130 inhibits USP9X, USP5, UCHL1, USP14, and UCH37 in liver and breast cancer [217,218,219], exhibiting anti-tumor activity by downregulating the anti-apoptotic protein MCL−1 and upregulating the pro-apoptotic protein p53 [220]. The anti-tumor activity increased when WP1130 was combined with bortezomib and administered to a lymphoma animal model [221].

A proteasome inhibitor, b-AP15, that targets 19S regulatory particle associated DUBs, including USP14 and UCHL5, showed anti-tumor activity. b-AP15 effectively inhibited the dissemination of an acute mouse model of C1498 leukemia, inhibiting tumorigenesis in multiple solid tumor mouse models including lung, colon, and breast carcinoma xenografts [207,213,222]. 

PX−478 is a potential CSC-targeted therapeutic drug molecule because of its inhibitory effect on HIF−1α signaling, which is hyperactivated in the hypoxic niches of CSCs [223]. C527 is an inhibitor of USP1 that upregulates p21 in mouse osteosarcoma cells, facilitating erythroid differentiation of leukemic cells [224]. 

Although DUBs are attractive targets, clinical development of small-molecule DUB inhibitors has been limited by several obstacles. Most DUBs have a catalytic cysteine active site that is optimal for inhibitor development, and those catalytic pockets are conserved. Screening for potent small-molecules that show selectivity among related DUBs with conserved catalytic pockets is a big challenge in developing DUB inhibitors for clinical use. Another big obstacle for DUB inhibitor screening is oxidative hydrolysis of the active-site cysteine. Because most DUBs transfer ubiquitin molecules through a reactive thiol group, the assays used to screen for inhibitors are prone to non-selective redox or alkylating false-positives [225]. Additionally, the complex mechanism of DUB enzymatic activity, which uses allosteric effects or substrate-mediated catalysis and several DUBs that switch between active and non-active conformations, complicates design and generation of specific DUB inhibitors. Moreover, nonspecific DUB inhibitors show nonspecific biological toxicity, such as accumulation of polyubiquitinated proteins and misfolded proteins and reduction in overall DUB activity that produces aberrant biological activity by DUB-regulated oncoproteins [226].

Thus, despite the growing attractiveness of DUBs as cancer targets, only a few DUB inhibitors have advanced through clinical trials for cancer therapy. VLX1570, an inhibitor of USP14, was advanced to a phase I/II clinical trial in combination with dexamethasone (NCT02372240) by the US Food and Drug Administration (FDA) in 2017 [227]. Another DUB inhibitor, mitoxantrone, is FDA-approved to inhibit USP11 and has advanced to phase I/II clinical trials to target diseases, such as relapsed acute myeloid leukemia, neoplasms, advanced recurrent or metastatic breast cancer, multiple sclerosis, and neuromyelitis optica [228,229]. Pimozide is an inhibitor of USP1 that is currently in a phase II clinical trial of patients with amyotrophic lateral sclerosis (NCT02463825, NCT03272503). Despite the hurdles in developing DUB inhibitors, recent technology advances, such as efficient ways to screen substrates and activity-based probes to monitor target engagement, and increased understanding of the physiological and pathophysiological roles of DUBs are facilitating clinical development of select DUB inhibitors.

## 4. Conclusions

Taken together, our findings indicate that CSCs are cancer cells that underlie tumor relapse, heterogeneity, and metastasis. Recent evidence identifies CSCs as the possible cells of origin in numerous cancers. CSCs can be regulated by stemness markers (Oct4, Sox2, KLF4, c-Myc, Nanog, and others), signaling pathways (Hh, Wnt, Notch, TGF/BMP, Hippo, and other pro-survival pathways), extracellular factors (hypoxia, Tumor-associated macrophages, ECM, and vascular niches), and other factors. To maintain their pluripotency and differentiation, CSCs have to maintain a balance between ubiquitination and deubiquitination. DUBs maintain CSC activity and stemness, implying their role in development of CSC-specific treatments. 

This article summarized the role of DUBs in regulating stem cell pluripotency, CSC-associated transcription factors, proteins, and signaling pathways and their prospective uses in cancer research. However, additional research is required to identify effective ways to eliminate CSCs. First, neither the precise role of DUBs in CSCs nor which particular cancers are primarily driven by CSCs have been elucidated with certainty. Second, only a small amount is known about the interaction between DUBs and cell-fate determinants, such as Oct4 and Nanog. Third, information is lacking about the roles of DUBs in regulating malignant transformation of healthy stem cells and the signaling pathways that regulate CSC stemness and malignant potential. Fourth, little is known about the role of DUBs and the crucial JAK/STAT, Notch, and PI3K signaling pathways in CSCs. Fifth, there is inadequate evidence supporting CSCs as mediators of tumor development, that therapeutic resistance is driven by the tumor microenvironment, and that DUBs are involved in those processes. 

Answering those questions will be crucial to development of therapeutic DUB inhibitors to limit tumor progression and relapse. The use of genome editing, epigenetics, and cellular metabolism studies should be considered in cancer therapy because DUB-mediated signaling and regulatory functions also contribute to CSC stemness. DUB inhibitors, natural products, and agents that specifically target CSCs can also be included in future studies. Although we have come a long way in our understanding of the signals that drive cancer growth and how those signals can be targeted, effective control of cancers remains a key scientific and medical challenge. Using conventional treatments to target CSCs is difficult because of epigenetics and aberrant signaling. Thus, an improved understanding of CSCs and their signaling pathways could improve modern therapeutic approaches for diverse cancers in the clinical setting.

## Figures and Tables

**Table 1:** Deubiquitinating enzymes associated with stem cell factors.

Factor	Associated DUB	Function	Representative CSC Type
Sox2	USP22, USP9X	Regulates transcription and growth of cancer cells	Brain, pancreatic, prostrate, lung
Nanog	USP21 USP3 USP16	Protein stabilization	Pancreatic, lung, glioma, ovarian
Oct 4	USP7 USP 44	Transcription factor Promotes differentiation	Melanoma, breast
c-Myc	USP36 USP37 USP22 USP28	Protein stabilization	Glioma, liver, lung
ID proteins	USP1	Protein stabilization	Glioma, osteosarcoma
SIRT1	USP22	Positive regulator	Colorectal
Klf4	MCPIP	Transcription factor	Pancreatic
Lin28	USP28	Promotes translation	Liver, oral
P53	USP2a, OTUB1 USP10 Ataxin−3 USP7 OTUD1 OTUD5 USP11	Protein stabilization	Osteosarcoma, glioma
PTEN	ATXN3 USP18 USP7	Transcription Protein stabilization Location Tumor suppression	Prostate, endometrial
c-met	USP8	Organ regeneration Promotes cancer	Liver, prostate
Bmi1	USP7 USP11	Embryonic development DNA damage repair Self-renewal	Glioma, lung, head, neck
LSD1/KDM1A	USP7 USP11 USP28	Protein stabilization	Breast, glioma
REST	USP7 USP15	Transcriptional repression	Glioma
PRC1	USP7 USP11 USP26	Protein stabilization	Prostate, ovarian, uterine
PRC2	BAP1	Regulation of gene expression	Prostate

**Table 2:** DUBs associated with stemness signaling pathways.

Signaling Pathway	CSC Fate	DUBs Involved	Functions
Hedgehog	Breast glioblastoma adenocarcinoma	USP21 USP 8	Cell growth, cell specialization, patterning of the body
Wnt signaling pathway	Glioma	USP4 USP8	Embryonic development
Notch	Ovarian	EIF3f USP9X USP12 CYLD USP28	Cell–cell communication
Hippo	Osteosarcoma	USP9X USP11	Modulates cell proliferation
TGF/BMP	Breast	OTUB1 USP9X	Embryonal development, cellular differentiation, hormone secretion, immune function

## Abbreviations

ALDH—aldehyde dehydrogenase; APC—adenomatous polyposis coli; ASCs—adult stem cells; BCL9—B-cell CLL/lymphoma 9; CML—chronic myeloma leukemia; CK1α—casein kinase 1α; CRC—colorectal cancer; CSC—cancer stem cell; DLL—delta-like proteins; Dvl—disheveled; DUBs—deubiquitinating enzymes; ECM—extracellular matrix; ESCs—embryonic stem cells; EMT—Epithelial-mesenchymal transition; GBM—glioblastoma multiforme; GICs—glioma-initiating cells; GSK−3b—glycogen synthase kinase−3b; HCC—Hepatocellular carcinoma; HIFs—Hypoxia-inducible factors; IAPs—inhibitors of apoptosis; ID—Inhibitors of DNA binding; IL—interleukin; iPSCs—induced pluripotent stem cells; JAG—jagged proteins; JAK/STAT—Janus-activated kinase/signal transducer and activator of transcription; KLF4—Krüppel-like factor 4; LATS—large-tumor-suppressor; LRP—low-density-lipoprotein-related protein; MIB—Mind Bomb; MSCs—Mesenchymal stem cells; MARK—microtubule affinity-regulating kinase; MM—multiple myeloma; NICD—Notch intracellular domain; NF-κB—nuclear factor-κB; PRRs—Pattern recognition receptors; PML—promyelocytic leukaemia; PI3K—phosphatidylinositol 3-kinase; PTEN—phosphatase and tensin homolog; PTM—post-translational modification; PKB—protein kinase B; SCID—severe combined immunodeficiency disease; Shh – Sonic hedgehog; Smo—Smoothened protein; TAZ—tafazzin; TEADs—TEA domain proteins; TGF-β—tumor growth factor-beta; TNFα—tumor necrosis factor alpha; TRAIL—tumor necrosis factor-related apoptosis-inducing ligand; VGLL4—vestigial-like family member 4; YAP—yes-associated protein 1.
